# MyD88 Mediates Colitis- and RANKL-Induced Microfold Cell Differentiation

**DOI:** 10.3390/vetsci9010006

**Published:** 2021-12-24

**Authors:** Yang Li, Shanshan Yang, Xin Huang, Ning Yang, Caiying Wang, Jing Zhao, Zhizhong Jing, Luc Willems, Guangliang Liu

**Affiliations:** 1 State Key Laboratory of Veterinary Etiological Biology, Lanzhou Veterinary Research Institute, Chinese Academy of Agricultural Science, Lanzhou 730046, China; iamliyang91@163.com (Y.L.); yangshanshan@caas.cn (S.Y.); 15739456858@163.com (X.H.); yangning01@caas.cn (N.Y.); caiying.wang@wur.nl (C.W.); 15099331402@163.com (J.Z.); jingzhizhong@caas.cn (Z.J.); 2 Molecular and Cellular Epigenetics (GIGA), University of Liege, 4000 Liege, Belgium; luc.willems@uliege.be; 3 Cell Biology and Immunology Group, Wageningen University and Research, P.O. Box 9101, 6700 HB Wageningen, The Netherlands

**Keywords:** colonic M cells, MyD88, colitis, DSS

## Abstract

Intestinal microfold (M) cells are critical for sampling antigens in the gut and initiating the intestinal mucosal immune response. In this study, we found that the oral administration of dextran sulfate sodium (DSS) and *Salmonella* infection induced colitis. In the process, the expression levels of M cell differentiation-related genes were synchronized with the kinetics of pro-inflammatory cytokines. Compared to wild-type (WT) mice, *MyD88*^−/−^ mice exhibited significantly lower expression levels of M cell differentiation-related genes. However, DSS induced colitis in *MyD88*^−/−^ mice but failed to promote the transcription of M cell differentiation related genes. Furthermore, the receptor activator of the Nuclear Factor-κB ligand (RANKL) upregulated the transcription of M cell differentiation related genes in murine intestinal organoids prepared from both WT and *MyD88*^−/−^ mice. Meanwhile, fewer changes in M cell differentiation related genes were found in *MyD88*^−/−^ mice as compared to WT mice. Hence, we concluded that myeloid differentiation factor 88 (MyD88) is an essential molecule for colitis- and RANKL-related differentiation of M cells.

## 1. Introduction

Microfold (M) cells are specialized epithelial cells located mainly at follicle-associated epithelium (FAE) [1] and villous epithelium [2] of the small intestine. These cells are also found at the epithelium of colonic mucosa [3] and nasopharynx-associated lymphoid tissue (NALT) [4]. M cells possess unique morphological features, such as irregular brush border, pocket structure, reduced glycocalyx, and microvilli [5]. These cells play an essential role in mucosal immune surveillance [6], conventionally known for capturing luminal antigens and delivering antigens particles to underlying immune cells. Many intestinal pathogens, such as murine norovirus [7,8], reovirus [8], *Salmonella typhimurium* (*S. typhimurium*) [9], and *Candida albicans* (*C. albicans*) [10] utilize M cells as an entry.

M cells differentiate from leucine-rich repeat-containing G-protein coupled receptor 5^+^ (Lgr5^+^) stem cells [11]. In this process, both the receptor activator of the Nuclear Factor-κB ligand (RANKL) and transcription factor Spi-B are involved [11,12]. Moreover, RANKL can even induce differentiation of enterocytes into M cells [13]. Besides, intestinal microbiota influence M cell differentiation. For instance, 7-day feeding in a typical animal house environment increases the number of M cells in specific pathogen-free (SPF) mice. Both *Salmonella enterica serovar typhimurium type III* and bacterial flagellin are capable of aising the number of M cells in the gut [14,15]. Additionally, the nuclear factor kappa B (NF-κB) can stimulate expression of tumor necrosis factor-α (TNF-α) which subsequently upregulates differentiation of M cells [3]. NF-κB production is mainly induced by activation of myeloid differentiation factor 88 (MyD88) or tumor necrosis factor receptor-associated factor 6 (TRAF6) [16,17]. Actually, the TRAF6-mediated NF-κB signaling pathway is involved in M cell development [18]. Therefore, we hypothesized that MyD88 may also participate in M cell differentiation.

In this study, we show that dextran sulfate sodium (DSS) and *Salmonella choleraesuis* (*S. choleraesuis*) cause severe colitis in mice. The colitis induced up-regulation of M genes related to differentiation of M cells and this is attenuated when MyD88 is knocked out. Meanwhile, DSS-induced colitis promotes the expression of glycoprotein 2 (GP2), a specific marker of M cells, in the colon of wild-type (WT) mice but not in *MyD88*^−/−^ mice. Furthermore, RANKL upregulates expression of M cell-specific genes in intestinal organoids and this is partly dependent on MyD88. Therefore, we concluded that MyD88 is a key molecule for regulating the expression of M cell specific genes in colon.

## 2. Methods and Materials

### 2.1. Animals and Related Materials

*MyD88*^−/−^ (B6.129P2(SJL)-*MyD88*^tm1.1Defr^/J, Stock No: 009088) mice were originally purchased from The Jackson Laboratory (Anthony L. DeFranco, University of California, San Francisco, CA, USA). All mice used in this study were 6-8 weeks old and were bred in the SPF facility of Lanzhou Veterinary Research Institute (LVRI). Dextran sodium sulfate (DSS) was purchased from HWRK Chemical Company. *S. choleraesuis* (Strain NO. CVCC79102) was purchased from the China Institute of Veterinary Drug Control. SPF WT C57BL/6 mice were purchased from the Experimental Animal Center of LVRI. All experimental procedures and animal care protocols were carried out by following Care and Use of Laboratory Animals of LVRI, Chinese Academy of Agricultural Sciences, China.

### 2.2. Inflammatory Models

For the DSS-induced inflammatory model, DSS 3% (M.W 40000, YEASEN, Shanghai, China) was added to the drinking water which was provided *ad libithum* for seven days. DSS-free drinking water was used provided to mice in the control group. For the *S. choleraesuis*-induced inflammatory model, *S. choleraesuis* were collected by centrifugation and re-suspended in PBS buffer. The mice in the infected group were gavaged with 10^4^ CFU of *S. choleraesuis* or with PBS as control. All mice were kept at room temperature under a constant 12-h light/dark cycle. Mice were weighed daily and sacrificed at day 8.

### 2.3. Histological Analysis

After euthanasia, mice colons were fixed in the FinePix tissue-fix solution (RightTech, Shanghai, China) for 24 h, dehydrated according to the standard protocol, and then embedded in paraffin. Then tissue blocks were sectioned and deparaffinized in xylene, stained with Haematoxylin and Eosin (H&E). Finally, double-blinded histological analysis was performed to evaluate the colitis level as described [19].

### 2.4. Western Blot Analysis

Total proteins were prepared from tissue samples of the colon using RIPA lysis buffer (Beyotime, Shanghai, China) with protease inhibitor. A total of 20 µg of protein was loaded in each lane for SDS-PAGE, transferred, and immunoblotted overnight at 4 °C with a primary antibody against GP2 (1:1000, MBL, Nagoya, Japan) and GAPDH (1:5000, Proteintech Group, Rosemont, IL, USA), and Goat anti-Rat IgG (OriGene Technologies, Rockville, MD, USA). Next, the membrane was incubated with a 1:5000 dilution of anti-Rab IgG secondary rabbit antibody (OriGene Technologies, Beijing, China). Finally, the immunoblots were developed with chemiluminescence detection reagents (Advansta, San Jose, CA, USA) [20,21].

### 2.5. Murine Intestinal Organoids Culture

Mice were sacrificed and the small intestine was collected. After a first wash in PBS buffer, the intestines were cut into pieces and dissociated with 2 mM EDTA buffer in 15 mL centrifuge tubes for 20 min at 4 °C. The samples were centrifuged at 300× *g* for 5 min to remove the EDTA buffer, and repeatedly pipetted with 5 mL PBS buffer. Next, crypts were collected through a 70 µm strainer (BD Biosciences, San Jose, CA, USA). Single crypts were centrifuged at 300× *g* for 5 min, and cultured in a 24-well tissue culture plate (Corning, New York, NY, USA) with Matrigel Matrix (Corning, New York, NY, USA) and Murine Intestinal Organoids Growth Medium (STEMCELL Technologies, Vancouver, BC, Canada). For the RANKL stimulation assay, organoids were stimulated with 100 ng/mL recombinant human RANKL (CST, Fall River, MA, USA) for one day (PBS buffer was used as control). The RANKL and mock control reagents were added daily.

### 2.6. RNA Extraction and Quantitative Real-Time PCR

After the mice were sacrificed, their colon was washed by cold PBS then collected. Total RNA was extracted from the colon samples using RNAiso reagent (TaKaRa, Beijing, China) and used for cDNA preparation using Honor II 1st Strand cDNA Synthesis SuperMix (Novogene, Beijing, China) with hexamer random primers. To evaluate the inflammation responses and differentiation of M cells within mouse colon, the relative expression levels of TNF-α, IL-1β, IL-6, GP2, Spi-B, RANK, TNF-α-induced protein 2 (Tnfaip2), and C-C motif ligand 9 (CCL9) were determined by RT–qPCR using ChamQ SYBR qPCR Master Mix (Vazyme, Nanjing, China). The primers used in this study are listed in Table 1.

### 2.7. Statistical Analysis

The data were presented as the means ± SEM. The significance between groups was analyzed by one-way analysis of variance or Student’s *t*-test with GraphPad Prism 7 software and *p* values.

## 3. Results

### 3.1. DSS Induces Colitis and M Cell Differentiation

**Figure 1:**
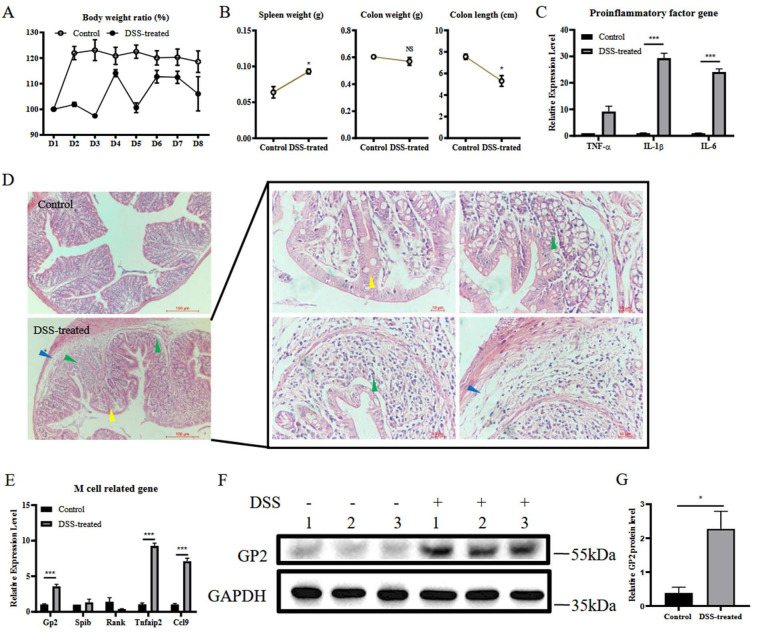
DSS induces colitis and M cell differentiation. WT mice in the DSS-treated group (*n* = 3) and control group (*n* = 3) were allowed to intake DSS-contained or normal water for 7 days freely. Their body weights were monitored daily (**A**). The mice were sacrificed on day 8, and their spleen weight, colon weight, and colon length were measured for assessing pathological changes caused by DSS treatment (**B**). Total RNA from colon samples was used to detect the mRNA levels of TNF-α, IL-1β, IL-6 by RT-qPCR (**C**). Partial colon samples were used for histological analysis to evaluate the colitis (**D**). Total RNA from colon samples was used to detect the mRNA levels of GP2, Spi-B, RANK, Tnfaip2, and CCL9 by RT-qPCR (**E**). The data were calculated using the 2^−∆∆Ct^ method. The GP2 expression levels in total protein from colon samples were evaluated by Western blot (**F**). Relative GP2 protein level was determined from the band intensity using ImageJ software (**G**). In all graphs, data are shown as ‘mean ± SEM’. Results were designated with: NS, *p* > 0.05; * *p* < 0.05; *** *p* < 0.001.

To investigate the relationship between M cell differentiation in the colon and colitis, a model of DSS-induced colitis was used. After oral administration of DSS for 7 days, the DSS-treated mice lost weight compared to the control group (Figure 1A). The weight of the spleen increased in DSS-treated mice, but weight and length of the colon were reduced (Figure 1B). The mRNA levels of the pro-inflammatory cytokines TNF-α, IL-1β, and IL-6 in the colon were quantified by RT-qPCR. DSS-treated mice had significantly higher mRNA levels of TNF-α, IL-1β, and IL-6 (Figure 1C). Histological changes, including infiltration of inflammatory cells (green arrow) in the basal layer and crypt, edema (blue arrow) and reduced mucus (yellow arrow), were observed in the colon of DSS-treated mice (Figure 1D). To assess the differentiation of M cells in the colon, mRNA levels of–genes related to M cell differentiation, including GP2, Spi-B, RANK, Tnfaip2, and CCL9, were measured by RT-qPCR. The results showed that the mRNA levels of GP2, Tnfaip2, and CCL9 from the colon of the DSS-treated group were significantly upregulated compared to the control (Figure 1E). The immunoblotting results showed that DSS treatment promoted the expression of GP2 (Figure 1F,G). These results indicated that oral administration with DSS successfully induced colitis, increased transcriptional level of genes related to M cell differentiation and GP2 expression.

### 3.2. S. choleraesuis Infection Induces Colitis and M Cell Differentiation

**Figure 2:**
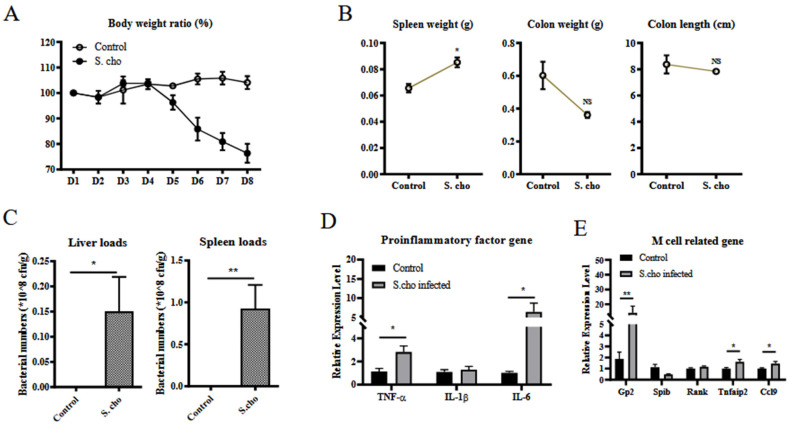

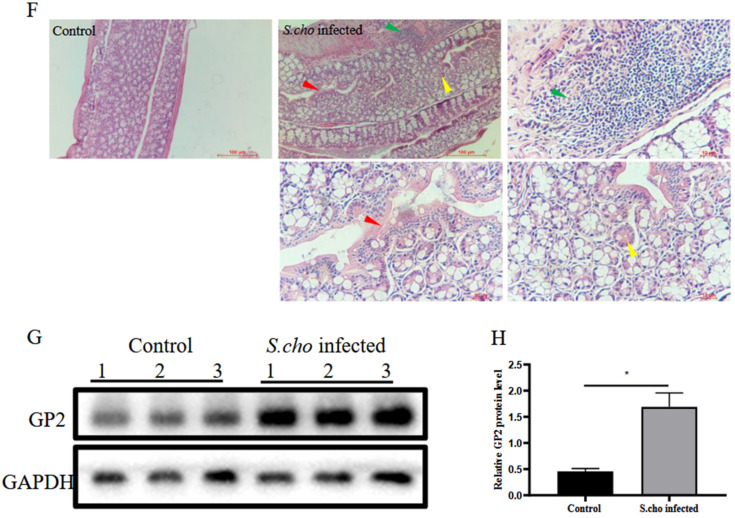
*S. choleraesuis* infection induces colitis and M cell differentiation. WT mice were gavaged with *S. choleraesuis*-PBS buffer mix (*S. choleraesuis*-infected group, *n* = 3) or normal PBS buffer (control group, *n* = 3). Their body weight was monitored daily (**A**). The mice were sacrificed on day 8, and the spleen weight, colon weight, and colon length were evaluated to assess the pathological changes caused by *S. choleraesuis* infection (**B**). The bacterial loads in the liver, spleen were analyzed (**C**). Total RNA from colon samples was used to detect the mRNA level of TNF-α, IL-1β, IL-6 by RT-qPCR (**D**). Total RNA from colon samples was used to detect the mRNA levels of GP2, Spi-B, RANK, Tnfaip2, and CCL9 by RT-qPCR (**E**). The data were calculated using the 2^−∆∆Ct^ method. Tissue samples from the colon were used for histological analysis for assessing colitis (**F**). The GP2 expression levels in total protein from colon samples were evaluated by Western-blot (**G**). Relative GP2 protein level was determined from the band intensity using ImageJ software (**H**). In all graphs, data are shown as ‘mean ± SEM’. Results were designated with: NS, *p* > 0.05; * *p* < 0.05; ** *p* < 0.01.

To further verify that colitis is related to differentiation of M cells in the colon, *S. choleraesuis* was administrated orally. Mice in the *S. choleraesuis*-treated group suffered weight loss compared to the mice in the control group (Figure 2A). Typical symptoms, such as increased spleen weight, and a reduction of colon weight and length, were observed in the S. choleraesuis-treated group (Figure 2B). Concomitantly, bacterial colonization in the liver and spleen was also shown (Figure 2C). The mRNA levels of TNF-α, IL-1β, and IL-6 in the colon were detected by RT-qPCR. The results illustrated that infection with the *S. choleraesuis* significantly upregulated the mRNA levels of TNF-α and IL-6 in the colon (Figure 2D). Pathological changes, including infiltration of inflammatory cells (green arrow) in the basal layer and crypt, fibrinous exudation (red arrow) and reduced mucus (yellow arrow), indicated *S. choleraesuis* induced colitis (Figure 2F).

To evaluate the differentiation of M cells, the mRNA levels of GP2, Spi-B, RANK, Tnfaip2, and CCL9 were measured by RT-qPCR. The result showed that the mRNA levels of GP2, Tnfaip2, and CCL9 in the colon from the *S. choleraesuis*-infected group were significantly higher than those in the control group (Figure 2E). Western blot analysis confirmed that *S. choleraesuis* infection induced GP2 expression (Figure 2G,H). Taken together, these data demonstrate that *S. choleraesuis* infection induces colitis. The colitis promotes the transcription of some genes related to M cell differentiation and the expression of GP2.

### 3.3. MyD88 Is a Critical Factor for Colonic M Cell Differentiation

**Figure 3:**
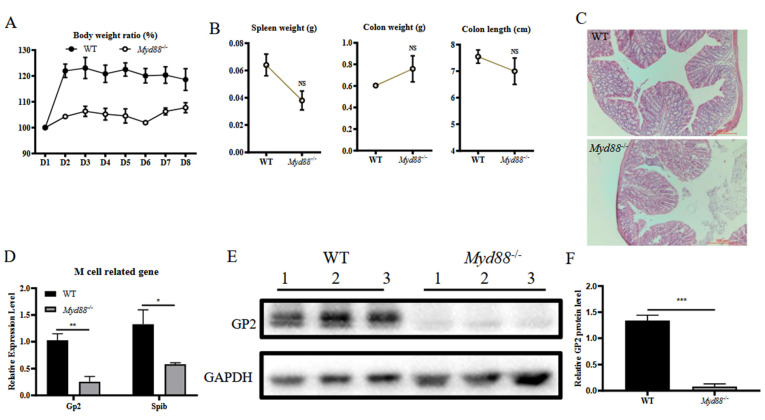
MyD88 is a critical factor for colonic M cell differentiation. The body weight of WT and *MyD88*^−/−^ mice (*n* = 3) were monitored for 8 days (**A**). After sacrificing, their spleen weight, colon weight, and colon length were measured (**B**). Partial colon samples were fixed for histological analysis (**C**). Total RNA from colon samples was used to detect the mRNA levels of GP2 and Spi-B by RT-qPCR (**D**). The data were calculated using the 2^−∆∆Ct^ method. The GP2 expression levels in complete protein from colon samples were evaluated by Western-blot (**E**). Relative GP2 protein level was determined from the band intensity using ImageJ software (**F**). In all graphs, data are shown as ‘mean ± SEM’. Results were designated with: NS, *p* > 0.05; * *p* < 0.05; ** *p* < 0.01; *** *p* < 0.001.

We speculated that MyD88 might play a role in the differentiation of M cells in the murine colon. To test this hypothesis, we compared WT and *MyD88*^−/−^ mice. During a 7-day housing period in the same environment, the bodyweight of both *MyD88*^−/−^ and WT mice remained stable (Figure 3A). On day 7, the weigt of the spleen was lower in *MyD88*^−/−^ mice than in WT mice, but weight and length of the colon were not significantly different (Figure 3B). Histological analysis of the colons did not reveal differences between *MyD88*^−/−^ and WT mice (Figure 3C). To investigate whether *MyD88*^−/−^ affects the differentiation of M cells in the colon, mRNA levels of genes related to M cell differentiation was detected by RT-qPCR while the GP2 protein expression was measured by western-blot. The results showed that the *MyD88*^−/−^ mice had significantly lower mRNA levels of GP2 and Spi-B, as well as GP2 protein levels compared to WT mice (Figure 3D–F). These results indicated that the lack of MyD88 restrained the transcriptional level of genes related to M cell differentiation in the colon and suggested that the MyD88 may play a critical role in regulating the differentiation of M cells in the colon.

### 3.4. MyD88 Is Involved in Colitis Induced M Cell Differentiation

**Figure 4:**
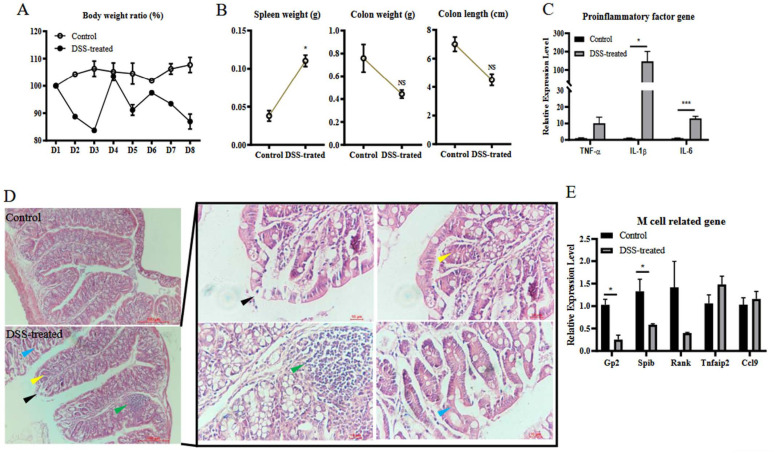
MyD88 is involved in colitis induced M cell differentiation. *MyD88*^−/−^ mice in the DSS-treated group (*n* = 3) and control group (*n* = 3) were allowed to intake DSS-contained or normal water for 7 days freely. The body weight of mice was monitored daily (**A**). The mice were sacrificed on day 8, and their spleen weight, colon weight, and colon length were measured to assess the pathological changes caused by DSS treatment (**B**). Total RNA from colon samples was used to detect the mRNA levels of TNF-α, IL-1β, IL-6 by RT-qPCR (**C**). Tissue samples from the colon were used for histological analysis to evaluate their colitis (**D**). Total RNA from colon samples was used to detect the mRNA levels of GP2, Spi-B, RANK, Tnfaip2, and CCL9 by RT-qPCR (**E**). The data were calculated using the 2^−∆∆Ct^ method. In all graphs, data are shown as ‘mean ± SEM’. Results were designated with: NS, *p* > 0.05; * *p* < 0.05; *** *p* < 0.001.

To explore whether MyD88 participates in the colitis-induced differentiation of M cells, *MyD88*^−/−^ mice were given DSS orally for 7 days. Compared to the control group, weight loss was observed in the DSS-treated mice (Figure 4A). Moreover, the DSS treatment group was associated with more severe clinical symptoms, characterized by increased weight of the spleen, decreased weight of the colon weight, and colon length (Figure 4B). Pathological changes, including inflammation of inflammatory cells (green arrow), exfoliation of epithelial cells (black arrow), crypt branching (blue arrow), decreased mucus (yellow arrow) (Figure 4D), and the higher mRNA levels of TNF-α, IL-1β, and IL-6 in the colon of the DSS-treated group (Figure 4C) were observed, indicating that DSS still induced colitis in *MyD88*^−/−^ mice. This also suggested that myD88 is not the only key to induction of inflammation. However, during this severe inflammatory response, expression of markers related to M cell differentiation decreased (GP2, Spi-B and Rank) or remained unchanged (Figure 4E). These results indicate that MyD88 was required in the colitis-induced up-regulation of genes related to M cell differentiation.

### 3.5. MyD88 Is Involved in RANKL-Induced M Cell Differentiation

**Figure 5:**
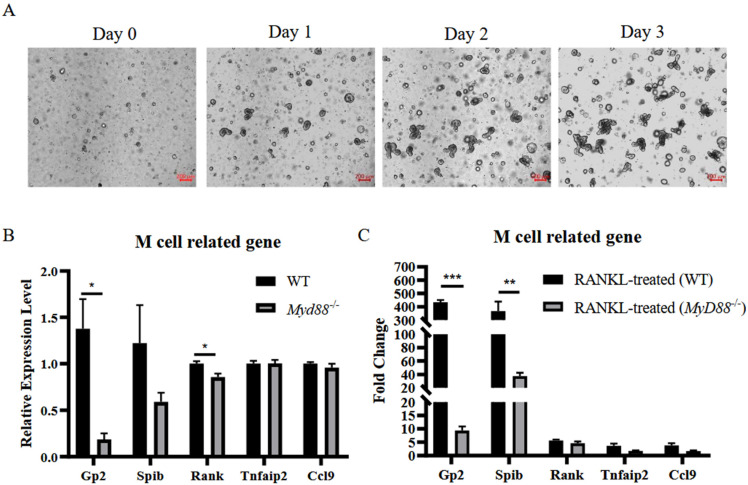
MyD88 is involved in RANKL-induced M cell differentiation. Murine intestinal organoids were established (**A**). Images of WT intestinal organoids were developed under a ZEISS Vert A1 microscope. Scale bar = 200 μm. Both WT and *MyD88*^−/−^ organoids were stimulated with human recombinant RANKL for one day (PBS was used as control) and then were collected for RNA isolation. The mRNA levels of representative M-cell related markers, GP2, Spi-B, RANK, Tnfaip2, and CCL9, in both WT and *MyD88*^−/−^ organoids, were detected by RT-qPCR (**B**). The fold change of these markers was compared after RANKL-treatment between WT and *MyD88*^−/−^ group (**C**). The data were calculated using the 2^−∆∆Ct^ method. In all graphs, data are shown as ‘mean ± SEM’. Results were designated with: * *p* < 0.05; ** *p* < 0.01; *** *p* < 0.001.

We hypothesize that MyD88 was also involved in RANKL-induced differentiation of M cells. To test this hypothesis, murine intestinal organoids were prepared for further study (Figure 5A). Expression of key M cell markers was evaluated by RT-qPCR in the organoids generated from WT and *MyD88*^−/−^ mice. The results showed that markers associated with M cell differentiation were down-regulated in *MyD88*^−/−^ mice (Figure 5B). This result supported that MyD88 was indispensable for differentiation of M cells in our previous results. Next, intestinal organoids from WT mice and *MyD88*^−/−^ mice were treated with RANKL 24 h before analysis. The results illustrated that RANKL is able to upregulate transcription of genes related to M cell differentiation in both WT mice and *MyD88*^−/−^ mice (Figure 5C), characterized as increased expression levels of GP2, Spi-B, RANK, Tnfaip2, and CCL9. However, the expression levels of M cell markers in *MyD88*^−/−^ mice were much lower than that in WT mice (Figure 5C). Taken together, these results indicate that MyD88 is also involved in M cell differentiation induced by RANKL.

## 4. Discussion

M cells are a potential route for promoting drug and oral vaccine delivery. M cell-dependent antigen transport was reported to have an essential function in alleviating colitis [22]. However, the knowledge about differentiation of M cells in the colon is limited. Research on the differentiation of M cells in the colon will help to better understand the role of M cells in maintaining intestinal homeostasis.

After a literature review, GP2 was used as marker for mature M cells [23]. The transcriptional level of GP2 is upregulated upon 2days of stimulation with RANKL [24]. Our study further proved that RANKL indeed induces differentiation of M cells in both WT and *MyD88*^−/−^ mice organoids, even though organoids of *MyD88*^−/−^ mice show lower expression levels of genes related to M cell differentiation compared to organoids from WT mice. Besides, intestinal microbiota could also regulate the differentiation of M cells [14]. As reported, *S. typhimurium* induces the differentiation of FAE enterocytes into M cells based on a type III effector protein SopB-dependent pathway [25]. In our study, we found that *S. choleraesuis*-induced colitis promoted differentiation of M cells. DSS-induced colitis also promoted the transcription of M cell specific genes. Therefore, we speculated that the upregulation of genes related to M cell differentiation r might be related to colitis. Moreover, we found that MyD88 plays a crucial role in both colitis- and RANKL-induced differentiation of M cells.

MyD88 is an essential adaptor molecule in the toll-like receptor signaling pathway, which is related to bacterial receptors [26] and homeostasis of intestinal microorganisms [27]. In the intestine, MyD88 also participates in regulating gut microbial ecology and maintaining homeostasis [28,29]. Here, we found that the differentiation of M cells is attenuated in both *MyD88*^−/−^ intestinal organoids and in the colon of *MyD88*^−/−^ mice as compared to the WT controls. These data suggested a potential crosstalk between MyD88-mediated signaling and differentiation of M cells. MyD88 has been proven to play a crucial role in regulating RANKL transcription induced by LPS and IL-1α [30]. In bone marrow stromal cells, MyD88 is also required for the up-regulation of RANKL transcription and down-regulation of osteoprotegerin (OPG) mRNA [31], further mediating differentiation of M cells in FAE [32]. These studies imply a relationship between the MyD88 mediated pathway and RANKL signaling. Hence, deletion of MyD88 may promote the transcription of OPG and potentially inhibit differentiation of M cells.

The differentiation of M cells may be regulated by a complicated signaling pathway related to the immune response and gut microbiota. Here, we highlight the crosstalk between the inflammatory response and M cell differentiation and demonstrate that MyD88 was required for colitis- and RANKL-induced up-regulation of genes related to M cell differentiation. Unfortunately, we were not able to perform a quantitative analysis regarding the differentiation of M cells from the colon by performing immunostaining for GP2 in tissue. Meanwhile, we found a low number of GP2+ M cells upon immunostaining of mice colons. Therefore, although colitis and RANKL induce the transcriptional level of the genes related to M cell differentiation, the detailed mechanism regulating differentiation of M cells isolated from the colon still needs further study.

## 5. Conclusions

The study showed that the transcription of genes related to differentiation of M cells from the colon was promoted in colitis induced by DSS and *S. choleraesuis*. Moreover, in *MyD88^−/−^* mice, DSS induced colitis but failed to upregulate mRNA levels of M cell specific genes in the colon. In contrast with the WT group, the significantly lower transcriptional level of M cell specific genes was detected in *MyD88*^−/−^ intestinal organoids and in colon of *MyD88*^−/−^ mice. At the same time, the deletion of MyD88 also attenuated RANKL stimulated upregulation of M cell specific genes in intestinal organoids. These data suggested a potential regulatory function of MyD88 in differentiation of M cells from the colon. To demonstrate a link between inflammation and M cell differentiation, further experiments will include blocking the TNF-α, IL-6 or IL-1β signaling, and evaluating its impact on differentiation of M cells.

## Figures and Tables

**Table 1:** Primers used in this study.

Target Gene	Primer/Probe	Sequence (5′–3′)
GAPDH	Forward	GCCTTCCGTGTTCCTACCC
	Reverse	TGCCTGCTTCACCACCTTC
Spi-B	Forward	GGGGGCCTTGACTCTA
	Reverse	CTCTGGGGGGTACACC
GP2	Forward	CCTGCGTTCTGACACTG
	Reverse	GCCGTGCAGGTTATCA
RANK	Forward	ATGCGAACCAGGAAAGT
	Reverse	TGCCTGCATCACAGACT
Tnfaip2	Forward	GTGCAGAACCTCTACCCCAATG
	Reverse	TGGAGAATGTCGATGGCCA
CCL9	Forward	GCCCAGATCACACATGCAAC
	Reverse	AGGACAGGCAGCAATCTGAA
TNF-α	Forward	TCAGTTCCATGGCCCAGAC
	Reverse	GTTGTCTTTGAGATCCATGCCATT
IL-1β	Forward	CCCTGAACTCAACTGTGAAATAGCA
	Reverse	CCCAAGTCAAGGGCTTGGAA
IL-6	Forward	TAGTCCTTCCTACCCCAATTTCC
	Reverse	TTGGTCCTTAGCCACTCCTTCC

## Data Availability

The data are available upon request from the authors.

## Abbreviations

CCL9: C-C motif ligand 9; DSS, dextran sulfate sodium; FAE, follicle-associated epithelium; GP2, glycoprotein 2; Lgr5, leucine-rich repeat-containing G-protein coupled receptor 5; M cells, microfold cells; MyD88, myeloid differentiation factor 88; NALT, nasopharynx-associated lymphoid tissue; RANK, receptor activator of NF-κB; RANKL, receptor activator of the Nuclear Factor-κB ligand; TNF-α, tumor necrosis factor-α; TRAF6, tumor necrosis factor receptor-associated factor 6.
